# Diagnosis, Rehabilitation and Preventive Strategies for Pudendal Neuropathy in Cyclists, A Systematic Review

**DOI:** 10.3390/jfmk6020042

**Published:** 2021-05-10

**Authors:** Rita Chiaramonte, Piero Pavone, Michele Vecchio

**Affiliations:** 1 Department of Biomedical and Biotechnological Sciences, Section of Pharmacology, University of Catania, 95123 Catania, Italy; 2 Department of Clinical and Experimental Medicine, University Hospital “Policlinico-San Marco”, 95123 Catania, Italy; ppavone@unict.it; 3 Rehabilitation Unit, “AOU Policlinico G.Rodolico”, 95123 Catania, Italy

**Keywords:** bicycling, pudendal neuralgia, rehabilitation, systematic review

## Abstract

This systematic review aims to provide an overview of the diagnostic methods, preventive strategies, and therapeutic approaches for cyclists suffering from pudendal neuropathy. The study defines a guide in delineating a diagnostic and therapeutic protocol using the best current strategies. Pubmed, EMBASE, the Cochrane Library, and Scopus Web of Science were searched for the terms: “Bicycling” OR “Bike” OR “Cyclists” AND “Neuropathy” OR “Pudendal Nerve” OR “Pudendal Neuralgia” OR “Perineum”. The database search identified 14,602 articles. After the titles and abstracts were screened, two independent reviewers analyzed 41 full texts. A total of 15 articles were considered eligible for inclusion. Methodology and results of the study were critically appraised in conformity with PRISMA guidelines and PICOS criteria. Fifteen articles were included in the systematic review and were used to describe the main methods used for measuring the severity of pudendal neuropathy and the preventive and therapeutic strategies for nerve impairment. Future research should determine the validity and the effectiveness of diagnostic and therapeutic strategies, their cost-effectiveness, and the adherences of the sportsmen to the treatment.

## 1. Introduction

Cyclists are particularly prone to trauma, infection, tumor, injury, and microtrauma related to their sport [1]. Cavernosal and dorsal arteries and pudendal nerve could be injured in many conditions, such as compression between the saddle and pubic bones or pubic symphysis during cycling or within the Alcock canal medial to the ischial rami [2,3].

While cycling, the body weight on the seat could compress nerves, vessels, or both. Repeated trauma to the perineum, prostatic disease in men, and pelvic pathology in females can favor the onset of the disorder [4,5]. According to Silbert et al. [5], the compression of the pudendal nerve could be related to the forward-leaning posture that presses perineum anteriorly to the ischial spine. According to Andersen and Bovim [6], if ischemia of the nerve by “compression posture” lasts less than 6 h, the block of nerve conduction is rapidly reversible, while if the ischemic period persists beyond 8 h, the recovery requires weeks. It could be related to a demyelinating block caused by direct pressure on perineum [6]. 

The pudendal neuralgia, caused by entrapment and compression of the pudendal nerve, is characterized by severe, sharp pain along the course of the pudendal nerve [7], genital numbness, erectile dysfunction (ED), and impotence [2,8]. 

Once afflicted, the cyclists are inclined to relapses; awareness of the problem could improve the adherence to prevention and therapeutic strategies [9]. Cyclists should pay close attention to any early warning symptoms and signs, such as pain, tingling, or numbness of the penis and/or perineum. Even in the absence of such symptoms, cyclists should follow several pieces of advice. 

Very few robust trials are present in the current literature, several of them not recent, despite the actuality of the disorder. This disabling condition related to musculoskeletal and neuropathic disorders often dictates to stop playing this sport, and this advice needs to be extended. New research could make substantial changes in the diagnostic path, and in taking charge. The therapeutically proposed solutions should not be directed to the suspension of cycling, but to timely treatment to achieve a complete recovery, rehabilitation from symptoms, and functional ergonomics. 

The systematic review aims to provide an overview of the diagnostic methods, preventive strategies, and therapeutic approaches for the cyclists suffering from pudendal neuropathy. The study defines a guide in delineating a diagnostic and therapeutic protocol using the best current strategies. Moreover, an update on the topicality of this disorder and on the disabling condition in those who practice this sport could increase the attention to the problem to obtain a dedicated field of interest and prevent the disorder.

## 2. Methods

### 2.1. Search Strategy

A systematic literature search on the preventive strategies and therapeutic approaches for the cyclists suffering from pudendal neuropathy was carried out. Pubmed, EMBASE, the Cochrane Library, and Scopus Web of Science were searched. The review was conducted from 1 May 2020 to 2 April 2021. 

### 2.2. Selection Criteria and Data Extraction

Two independent reviewers (R.C. and M.V.) screened articles by title and abstract for the following key terms: “Bicycling” OR “Bike” OR “Cyclists” AND “Neuropathy” OR “Pudendal Nerve” OR “Pudendal Neuralgia” OR “Perineum”. We included original articles (case reports, case series, observational and prospective studies) in English on prevention and therapeutic strategies for pudendal neuropathy in healthy cyclists. Only published data were included. We excluded animal studies and studies with participants who had no peripheral perineum neuropathy and those different from cyclists. We also excluded all duplicate studies. 

The systematic review was executed according to the PRISMA checklist [10] and the PICOS criteria [11] (population, intervention, comparison, outcome, and study design). As shown in Table 1, the participants were cyclists, and the interventions were based on prevention and rehabilitative or pharmacologic treatment. The comparator could be any comparator, and the outcomes included clinical assessments, diagnostic scales, and nerve conduction studies, as well as radiologic imaging. 

## 3. Result 

### 3.1. Description of the Studies

From 1975 to 2021, the database search identified 14,602 articles. After the titles and abstracts were screened, the reviewers analyzed 41 full texts. Additionally, the reference lists of relevant articles were screened for any other eligible articles to include for review. The studies’ eligibility was assessed independently. 

**Figure 1:**
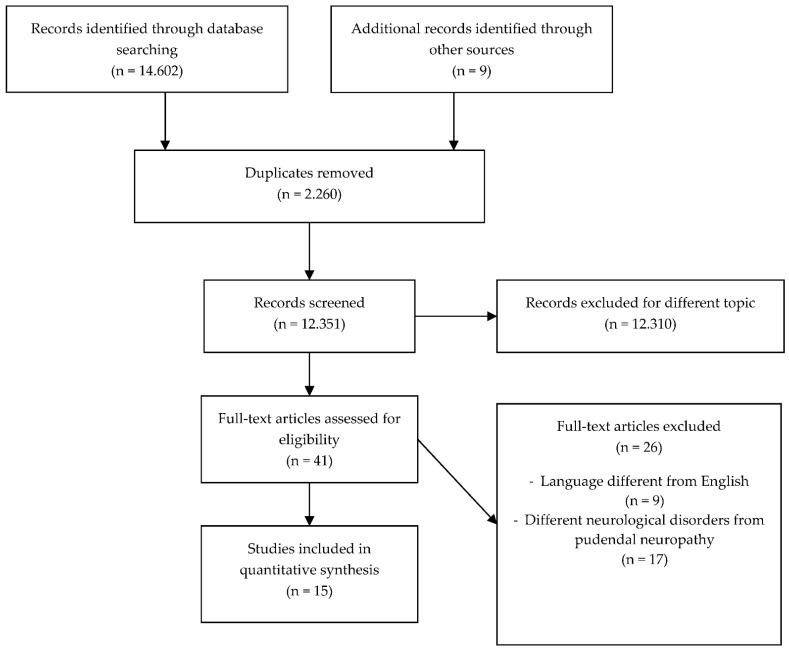
Flowchart of the process of literature search and extraction of studies meeting the inclusion criteria.

Twenty-six articles were excluded for the following reasons: 9 did not use the English language, 17 examined different neurological disorders from pudendal neuropathy. Figure 1 shows the number of studies produced at each stage of the search. A total of 15 articles were considered eligible for inclusion (Figure 1 and Table 1).

### 3.2. Variations of Experimental Conditions across the Studies

The methods used in each of the 15 selected articles for the prevention and treatment of peripheral neuropathy of perineum in cyclists were described. The study groups were homogeneous for relevant general clinical features, such as sport practice and localization of the lesion, but not for clinical presentation, duration of symptoms, miles before starting of symptoms, types of diagnostic measures, severity of symptoms, and therapy (Table 1). 

### 3.3. Summary of Findings

#### 3.3.1. Diagnostic Examination

The systematic review showed preventive and therapeutic approaches for peripheral neuropathy in cyclists and all the diagnostic methods used in the current literature. 

Clinical evaluation related to the sport is considered enough for the diagnosis of pudendal neuropathy due to cycling [3,5,6,9,13,17,19].

Several scales and instrumental diagnostics were used to diagnose the severity of the disorders: Pain Intensity Scale [8], International Index of Erectile Function [14], Dennerstein Personal Experience Questionnaire (SPEQ) [15], Female Sexual Distress Scale (FSDS) [15]. The diagnostic methods included nerve conduction studies and electromyography [13,18], radiologic diagnosis with ultrasonography and doppler waveforms [2,12,13], computed tomography [4], magnetic resonance imaging (MRI) [2], and diagnostic arteriography [12].

#### 3.3.2. Bike Elements Related to Peripheral Neuropathy

The area of contact between the bicyclist and the bike is the cause of nerve compression. Comfortable characteristics of the bicycle and practical recommendations are shown in Table 2. 

##### Bike

Bicycle characteristics associated with an increased risk of erectile dysfunction included a mountain bicycle compared to a road bicycle [14]. 

##### Seat

Prolonged sitting on a hard, narrow, and upward-tilted seat contributes to the development of impotence [3]. The narrow saddle is associated with a significant reduction in penile blood flow and could be a source of blunt perineal trauma with consequent erectile dysfunction [20]. The upward-tilted seat places greater pressure on the perineum [21]. 

The use of cut-out saddles could increase the pressure along the area of the pudendal nerves and vessels [22], with a higher risk of ED compared with a traditional saddle shape, particularly in those who had perineal numbness [14].

According to Carpes et al. [23], the seat pressure was not different between men and women. Using plain saddles, the men’s average seat pressure increased as the workload increased. Using a holed saddle, the mean pressure increased as the workload increased both in men and in women [23]. This study [23] was not included in the systematic review because the tested cyclists did not report any symptoms. 

##### Handlebars

A height of the handlebars parallel with or higher than the saddle could increase the risk of pudendal neuropathy compared to handlebar height lower than the saddle [14]. A height of handlebars lower than the saddle could increase vibratory thresholds and cause decreased genital sensation in the anterior vagina and labia [17].

The use of triathlon bars causes cyclists to move forwards the body with an excessive pressure on the perineum and compression of the pudendal nerve [5].

### 3.4. Sex Influence

Most of the articles analyzed nerve impairment [2,3,4,5,6,8,14] and ischemic neuropathy condition [9,12,13,18,19] in men.

Only a few articles analyzed the corresponding conditions in women [15,16,17]. There is an association between bicycling and decreased genital sensation in competitive women bicyclists, even if negative effects on sexual function and quality of life were not apparent in young, healthy premenopausal cyclists [15]. A correlation between bicycle set-up and neurological impairment was considered in female cyclists [17]. A study suggests that cut-out and narrow saddles could negatively affect saddle pressures in female cyclists [16]. An association was highlighted between bicycling and decreased genital sensation in competitive women bicyclists [15]. Correcting modifiable risks factors for pelvic floor damage may serve as the most important next step in enhancing riding safety in women cyclists [17].

## 4. Discussion 

This systematic review gives an overview of all diagnostic methods used and preventive and therapeutic strategies essential for cyclists to avoid pudendal neuropathy. The study describes the musculoskeletal and neuropathic disorders caused by careless physical exercise contrary to what is generally reported in the literature, namely, the role of physical exercise to prevent and treat musculoskeletal disorders. The role of correct training in the musculoskeletal and neuropathic disorders is essential to avoid traumatic and overuse-related symptoms. The execution of the athletic gesture should improve performance and not cause related pathologies.

### 4.1. Diagnostic Approach

Pudendal neuralgia is a diagnosis of exclusion. The multidisciplinary team of Nantes, France and Francophone Perineal Electrophysiology members in 2008 drew up the diagnostic criteria [27].

Clinical examination supports the definitive diagnosis [3,5,6,9,13,17,19]. 

Several scales were used to diagnose the severity of the disorders. Durante et al. [8] used the Pain Intensity Scale, Dettori et al. [14] used the International Index of Erectile Function [28], Guess et al. [15] used the Dennerstein Personal Experience Questionnaire (SPEQ) [29] and the Female Sexual Distress Scale (FSDS) [30]. 

Four studies assessed the neuropathy compression with radiologic imaging [2,4,12,13]. Ricchiuti et al. [18] performed the electromyography that evidenced a bilateral pudendal nerve injury. Guess et al. [15,16] determined the genital vibratory thresholds (VTs), but they did not find any correlations between VTs and miles biked per week, duration of riding, or BMI (body mass index) of cyclists [15]. Partin et al. [17] described a significative association between increased VTs and decreased genital sensation in the anterior vagina and labia.

### 4.2. Cautions to Avoid Peripheral Neuropathy

Several articles highlight a spontaneous resolution with rest [2,5,19] and reduction of sport activity [18], modifications of bike components [3,6,9,14], and following a rehabilitation program [8,23,24].

The characteristics of the bike could influence the symptomatology related to pudendal nerve compression. Dettori et al. [14] suggested to choose a road bicycle instead of a mountain bicycle. Specific size and shape of saddle, handlebars, and the duration of rest period affect the onset and the severity of the compression neuropathy. These limitations of activity are often less accepted by athletes [6].

#### 4.2.1. Breaks and Rest

The changes of the riding position during the training can alleviate symptoms [5,9]. During the training, frequent breaks, shifting to a higher gear, and standing on the pedals periodically can take pressure off the genital area [9,26]. Several authors recommend a period of rest during the race [3,9,26], exactly 20–30 s of rest every 20 min [23,24].

Reduction of activity [18] or rest [19] can reduce the symptoms after their onset. 

#### 4.2.2. Seat Arrangement

Bicycles should be fitted properly, and the saddle should be adjusted to the proper height and angle to avoid nerve compression. The US Army equestrian saddle has a slot in the center so that there is no pressure against the penis [9]. The therapeutic recommendations include a greater and wider seat padding, an absent or flexible nose on the saddle, a gel saddle, a more downward seat position or slighter tilt downwards to avoid anterior compression [3,9,23,24]. The seated weight should set down on the ischial tuberosities. The seated height should permit a slight flexion during pedaling at the lowest point of the pedal. Reducing the pressure on the perineum appears to be the solution, because in the cyclists the repetitive sliding of the fascia lata could decrease penile perfusion [25].

A saddle without a cut-out could help in the cases of perineal numbness [14].

Alongside wide and padded saddles, padded biking shorts increase comfort and protect the perineal soft tissue more than the other seat designs [26].

#### 4.2.3. Handlebars

In addition to adjusting the seat, cyclists should attend to the handlebar position [31]. Maintaining height of the handlebar lower than the saddle could prevent nerve compression [14].

### 4.3. Rehabilitation and Physical Exercises

A specific program of exercises could help weight loss if necessary as overweight could worsen nerve compression. Specific exercises are important for making adjustments in technique and improving the body posture to a more upright position. Stretches and rest for 3–10 days often promote recovery [23,24]. 

Durante et al. [8] presented the Active Release Technique (ART) for the treatment of symptoms related to pudendal nerve entrapment [32]. The practitioners apply tension to muscles and the patients actively contract and shorten the muscles and then stretch and tense them [32].

### 4.4. Invasive Treatment

Treatment is related to the degree of discomfort and symptoms. Conservative measures are often enough. In cases of severe, intractable discomfort and dysfunction, more aggressive and invasive treatment is necessary.

Calvillo et al. [4] used a CT-guided pudendal nerve block to temporarily relieve long-standing perineal and scrotal pain in a cyclist. Surgical decompression of the pudendal nerve could be a therapeutic option in cyclists for whom there was only temporary relief after the nerve block. 

Several cases of high-flow priapism as a result of acute bilateral perineal trauma sustained during bicycling have been reported [12,33]. They were treated successfully with percutaneous arterial embolization [12,33].

### 4.5. Pharmacological Treatment

Symptomatic drugs might have beneficial effects, anti-inflammatory and nerve pain medications or vasodilator drugs may be used in cases of ischemic neuropathy.

Gabapentin 300 mg daily for 6 months did not yield any success [4]. 

For the pudendal nerve block, under CT guidance, injection of 4 mL of lidocaine with 1 mL of triamcinolone (40 mg) relieves the symptoms for a longer period of time [4].

## 5. Study Limitations 

In this systematic review, most studies are of low to moderate quality. Several studies are case reports or have very small sample sizes, leading to questions regarding their statistical power.

## 6. Conclusions

This research highlights all the preventive and therapeutic strategies towards obtaining a guide for those who treat, train, and support cyclists with pudendal neuropathy. Increased attention to the execution of the athletic gesture is essential to obtaining good sport results, and especially to avoiding the possibility of training becoming the cause of musculoskeletal and neuropathic disorders.

New bicycle designs pay attention to preserving the perineum, avoiding nerve compression, reducing perineal pressure, and preventing impingement of the pudendal nerve. However, nerve compression could be present, and its diagnosis and treatment are necessary. 

The symptomatology related to pudendal neuropathy could affect experienced and novice cyclists. Cyclists could develop a less severe disorder by maintaining better posture on the bike and adhering to the advice on preventive measures. A conservative treatment permits the recovery, rarely requiring invasive treatment. 

Although the studies included in the systematic review on this topic present a moderate or low level of evidence, they could lead to new original and innovative leaps in the study the potential problem in more detail. Despite the range of tools available, robust trials are lacking, and the diagnostic and therapeutic approaches are often different. More research is needed to determine the measurements of treatment adherence and cost-effectiveness, the best diagnostic methodologies, and preventive and therapeutic strategies, to delineate a definitive diagnostic and therapeutic protocol, including preventive tools, such as improved bike models and new bike elements.

## Figures and Tables

**Table 1:** Characteristics and outcomes of studies included in the systematic review.

Authors	Study Design	Participants	Beginning of Symptomatology	Symptoms	Diagnosis	Treatment	Outcomes
Andersen 1997 [6]	Observational study	160 cm, 37.5 ± 10.9 y	After 540 km	Penile numbness or hypaesthesia, ED after the tour for h to m.	Clinical diagnosis	Besides changing the body position on the bike, restricting the training intensity and taking ample pauses may also be necessary in prolonged and vigorous bicycle riding to prevent damage to peripheral nerves.	22% reported numbness, 13% impotence. It lasted for more than 1 week in 11, and for more than 1 month in 3 participants.
Bond 1975 [9]	Case series	22 c	After 40 miles or more	Numb penis during and after a ride.	Clinical diagnosis	Tilting the peak of the saddle downward, shifting their weight on the saddle, stopping to rest, and shifting to a higher gear and standing up to pedal.	It is a benign disorder with spontaneous resolution usually occurring overnight.
Calvillo 2000 [4]	Case report	1 c, 52 y	After 10 min	Anoperineal pain from 2 y.	CT	Gabapentin 300 mg daily for 6 months without any success. Diagnostic bilateral pudendal nerve block under CT guidance, injecting 4 mL of lidocaine with 1 mL of triamcinolone (40 mg)	The use of CT to guide the procedure allowed precision in performing the procedure and in making a differential diagnosis.
De Rose 2001 [12]	Case report	2 c, 31.5 y	1 c: immediately after a trauma	ED for 2 m.	Intracorporeal blood gas analysis, color Doppler ultrasonography, and selective pudendal arteriography	Embolization of the fistula with gelatin sponge	Cycling should be considered a possible risk factor for arterial priapism as it is for urethritis, prostatitis, hematuria, testicular torsion, scrotal and penile numbness, and erectile dysfunction.
Desai 1989 [13]	Case report	1 c, 27 y	After 32 km bicycle race	Ipoaesthesia, loss of erections for about three weeks.	Doppler, EMG	-	Description of the case report.
Dettori 2004 [14]	Prospective study	463 c	After 320 km race	Perineal numbness during the ride, erectile dysfunction for 8 m.	International Index of Erectile Function	Cyclists on a long-distance ride may be able to decrease the risk of erectile dysfunction by riding a road bicycle instead of a mountain bicycle, keeping handlebar height lower than saddle height, and using a saddle without a cutout if perineal numbness is experienced.	Associations between erectile dysfunction risk and riders.
Durante 2010 [8]	Case report	1c, 41 y	After 6–11 h per week, 3 days a week of training	Penis pain 12–24 h after long distance cycling and pain after sexual intercourse. Hyperalgesia was found during palpation of the lesser sciatic notch and the obturator internus muscle.	Pain intensity scale	Treated twice a week for 4 w with ART obturator internus muscle protocol.	Diagnosis and treatment of pudendal nerve entrapment.
Goodson 1981 [3]	Case report	1 c, 46 y	After a 2-day, 180-mile ride	Diminished sensitivity to light touch along the penile shaft, numbness for 4 w.	Clinical assessment	Added seat padding or more downward seat slanting is a therapeutic recommendation.	Pudendal compression between bike seat and pubic symphysis can cause impairment of sexual response.
Guess 2006 [15]	Observational study	48 c, 22 hc, 33 y	Average of 28.3 ± 19.7 miles/d, 3.8 ± 1.5 d/w, for an average of 2.1 ± 1.8 h/r	Normal sexual function.	VTs, SPEQ, FSDS	-	Increasing VTs at the clitoris, anterior vagina, and urethra were associated with age. In bicyclists, there were no correlations between VTs and miles biked per week, duration of riding, or BMI
Guess 2011 [16]	Case series	48 c, 35.98 ± 6.90	99.24 ± 74.11 miles/w	Pain, numbness, and edema of pelvic floor structures.	VTs	-	Cut-out and narrower saddles negatively affect saddle pressures in female cyclists
Oberpenning 1994 [2]	Case reports	2 c -	-	Numbness for 4–6 w.	Sonography of abdomen, prostate and testes, MRI of pelvis and lumbar spine, Doppler sonography	The symptoms in the 2 patients spontaneously resolved after 4 and 7 weeks, respectively, without specific medical therapy.	Description of intermittent genital hypesthesia that occurred in cyclists after long-term bicycle riding.
Partin 2012 [17]	Observational study	c, 22 runners	>10 miles/w	62% genital numbness, tingling or pain	Clinical diagnosis, VTs	Modifying the handlebar level	Correlation between bicycle set-up and neurological compromise in women cyclists.
Ricchiuti 1999 [18]	Case report	1c, 44 y	3000 m/y	ED, numbness	EMG evidence of bilateral pudendal nerve injury.	C decreased bicycling from 3000 to approximately 1500 miles per year due to the persistent symptoms.	This condition may be associated with male ED if the penile blood supply is compromised.
Silbert 1991 [5]	Case reports	2 c	A: after switching to triathlon bars and a narrow firm seat. B: after being hit by a car and sustained a perineal injury.	Penile numbness	Clinical assessment	A: Symptoms resolved after the subject returned to traditional drop bars and a softer saddle. B: After a period of not cycling, his symptoms resolved completely.	Pudendal nerve pressure neuropathy can result from prolonged cycling, particularly when using a poor riding technique.
Solomon 1987 [19]	Case report	1 c, 55 y11	After beginning to use a stationary bike.	Penile numbness and ED	Clinical diagnosis	Resolved once he stopped riding.	A relationship between sexual dysfunction and bicycling may be more common than formerly suspected.

Cyclists, c; years old, y; erectile dysfunction, ED; electromyography, EMG; observational study, OS; hours, h; weeks, w; months, m; days, d; vibratory thresholds, VTs; Dennerstein Personal Experience Questionnaire, SPEQ; Female Sexual Distress Scale, FSDS; magnetic resonance imaging, MRI; Active Release Technique, ART.

**Table 2:** Comfortable characteristics of the bicycle and practical recommendations.

Practical Recommendations	Characteristics of Bike Parts and Practical Strategies	References
Bicycle parts: seat	Soft, wide	[5]
	Horizontal and not inclined seat	[23,24]
	Absent or flexible nose on the saddle	[23,24]
	Saddle without a cut-out	[14,16,22]
Bicycle parts: handlebars	Handlebar height lower than the saddle	[14]
	Avoiding triathlon bars	[5]
Sportswear	Padded biking shorts	[25]
Rest	Reduction of sport activity	[2,5,12,20]
Advice	Frequent breaks	[2,5,9,18,19,23,24,26]
	Shifting to a higher gear, and standing on the pedals periodically	[9,26]
Rehabilitation program	Specific exercises for adjustments in technique and body posture to a more upright position, stretching	[8,23,24]

## Data Availability

Not applicable.
